# The Inhibition of SARS-CoV-2 and the Modulation of Inflammatory Responses by the Extract of *Lactobacillus sakei* Probio65

**DOI:** 10.3390/vaccines10122106

**Published:** 2022-12-09

**Authors:** Irfan A. Rather, Lee-Ching Lew, Majid Rasool Kamli, Khalid Rehman Hakeem, Jamal S. M. Sabir, Yong-Ha Park, Yan-Yan Hor

**Affiliations:** 1 Department of Biological Sciences, Faculty of Science, King Abdulaziz University, Jeddah 21589, Saudi Arabia; 2 Center of Excellence in Bionanoscience Research, King Abdulaziz University, Jeddah 21589, Saudi Arabia; 3 Probionic Corporation Jeonbuk Institute for Food-Bioindustry, 111-18, Wonjangdong-gil, Deokjin-gu, Jeonju-si 54810, Republic of Korea; 4 Princess Dr. Najla Bint Saud Al-Saud Center for Excellence Research in Biotechnology, King Abdulaziz University, Jeddah 21589, Saudi Arabia; 5 Department of Public Health, Daffodil International University, Dhaka 1341, Bangladesh; 6 Department of Biotechnology, Yeungnam University, 280 Daehak-Ro, Gyeongsan 38541, Republic of Korea; 7 PYH Lab, Yeungnam University, 280 Daehak-Ro, Gyeongsan 38541, Republic of Korea

**Keywords:** *Lactobacillus* *sakei*, PROBIO65, SARS-CoV-2, COVID-19, viral replication, ROS, ERK, inflammatory

## Abstract

In the three years since the first outbreak of COVID-19 in 2019, the SARS-CoV-2 virus has continued to be prevalent in our community. It is believed that the virus will remain present, and be transmitted at a predictable rate, turning endemic. A major challenge that leads to this is the constant yet rapid mutation of the virus, which has rendered vaccination and current treatments less effective. In this study, the *Lactobacillus sakei* Probio65 extract (P65-CFS) was tested for its safety and efficacy in inhibiting SARS-CoV-2 replication. Viral load quantification by RT-PCR showed that the P65-CFS inhibited SARS-CoV-2 replication in human embryonic kidney (HEK) 293 cells in a dose-dependent manner, with 150 mg/mL being the most effective concentration (60.16% replication inhibition) (*p* < 0.05). No cytotoxicity was inflicted on the HEK 293 cells, human corneal epithelial (HCE) cells, or human cervical (HeLa) cells, as confirmed by the 3-(4,5-dimethylthiazol-2-yl)-2,5-diphenyl-2H-tetrazolium bromide (MTT) assay. The P65-CFS (150 mg/mL) also reduced 83.40% of reactive oxidizing species (ROS) and extracellular signal-regulated kinases (ERK) phosphorylation in virus-infected cells, both of which function as important biomarkers for the pathogenesis of SARS-CoV-2. Furthermore, inflammatory markers, including interferon-α (IFN-α), IFN-ß, and interleukin-6 (IL-6), were all downregulated by P65-CFS in virus-infected cells as compared to the untreated control (*p* < 0.05). It was conclusively found that *L. sakei* Probio65 showed notable therapeutic efficacy in vitro by controlling not only viral multiplication but also pathogenicity; this finding suggests its potential to prevent severe COVID-19 and shorten the duration of infectiousness, thus proving useful as an adjuvant along with the currently available treatments.

## 1. Introduction

Coronavirus Disease-2019 (COVID-19), rapidly recognized as a pandemic, triggered a global public health emergency with a death toll of approximately 15 million between 2020 and 2021. The predominant clinical manifestations exhibited by COVID-19 patients are related to respiratory and gastrointestinal tract disorders. The virus that causes the disease—SARS-CoV-2—is an enveloped, single-stranded RNA virus that transmits via aerosols (respiratory droplets suspended in the air) when prolonged face-to-face contact is maintained, or when a person is exposed to contaminated surfaces [1]. The entry of SARS-CoV-2 into host cells triggers an antiviral inflammatory response, consisting of both the innate and adaptive immune responses. In severe cases, it can induce sepsis, a condition marked by life-threatening organ dysfunction due to a dysregulated immune response (cytokine storm) [2]. Although vaccination remains the forefront prevention and treatment strategy for COVID-19, the virus continues to evolve and mutate rapidly. Therefore, managing SARS-CoV-2 infection requires exploring effective multidimensional therapeutic approaches, especially in underdeveloped countries where vaccine regimens are difficult to procure and implement.

Previous studies have established that a dysbiotic intestinal microbiome can cause pulmonary dysfunction by modulating the host’s immune response through the gut–lung axis [3]. In parallel, some studies have highlighted that COVID-19 patients display gut dysbiosis, with a depleted population of beneficial gut microbes such as *Lactobacillus* and *Bifidobacterium*, as well asdepleted gut diversity, and an abundance in populations of pathogens [2]. A study by Nobel et al. found that COVID-19 patients with gastrointestinal problems generally experienced SARS-CoV-2 infection for extended periods [4]. In addition, the depletion of beneficial gut microbial populations contributes to a dysregulated immune response, which can be life-threatening [5]. Therefore, researchers turned to probiotics as an adjuvant therapeutic strategy to address intestinal dysbiosis and to alleviate COVID-19 symptoms. In a study involving COVID-19 patients with severe pneumonia, the administration of probiotics (*Lactobacillus rhamnosus* GG, live *Bacillus subtilis*, and *Enterococcus faecalis*) significantly reduced the need for ventilators [6].

Interestingly, a study by Bousquet et al. pointed out that the low COVID-19 death rates in Eastern Asia and Central Europe may be attributed to the high level of consumption of fermented food, particularly fermented vegetables, which is common to these regions [7]. The study also postulated that this effect could be due to the high concentration of precursor molecules for nuclear factor-2 (Nrf2) in fermented cabbage [7]. Nrf2 is a potent antioxidant that can counteract the oxidative stress caused by SARS-CoV-2 upon the virus’s attachment to the angiotensin II receptor (ACE2) during its entry into the host cells [7]. Fermented vegetables are suitable reservoirs for lactic acid bacteria, primarily lactobacilli, and many of these strains have a long history of use and are commercialized as probiotics.

Due to their numerous therapeutic implications for intestinal health and other areas related to the gut–organ axis, probiotics can dynamically regulate the hosts’ health [8,9]. They are generally regarded as safe (GRAS) and have also been shown to produce many bioactive compounds with antiviral activities [10,11]. In our previous study, we reported the potential of bacteriocins from *L. plantarum* Probio-88 (plantaricin E and F) in the inhibition of SARS-CoV-2 replication and in immune regulation, using an in vitro and in silico approach [12]. In this study, we intend to further evaluate the effects of other probiotic strains, which do not produce plantaricins E and F, on the pathogenesis of SARS-CoV-2. 

The strain *Lactobacillus sakei* Probio65, which is isolated from Kimchi, a type of fermented cabbage from Korea, has previously been reported to exert broad antimicrobial properties, as well as displaying antiviral activity against avian influenza virus (H9N2), immune modulation, and the ability to reduce inflammation [13,14]. The patented strain is also recognized as a health-functional ingredient by the Ministry of Food and Drug Safety (MFDS) in Korea, as it has immunomodulatory effects on hyperimmune sensitivity. Based on the previous evidence that supports the immune-regulating benefits and antiviral properties of *L. sakei* Probio65, our research used an in vitro approach to determine whether the strain could affect the SARS-CoV-2 antiviral immune response.

## 2. Materials and Methods

### 2.1. Bacterial Strains, Media, and Growth Conditions

The bacterium used in this study was *L. sakei* Probio65, a patented strain previously isolated from Kimchi, a traditional Korean fermented cabbage. The bacteria were cultured at 37 °C for 18 h in modified de Man–Rogosa–Sharpe (MRS), as previously reported [12]. The strain was preserved in 25% glycerol (−45 °C) and activated three consecutive times using 1% inoculum before any experimental analysis. All chemicals mentioned herein were obtained from Sigma-Aldrich, St. Louis, MO, USA, unless otherwise stated.

### 2.2. SARS-CoV-2 Virus

The SARS-CoV- 2 strain used in this study was obtained from an oropharyngeal swab specimen of a patient who had tested positive for coronavirus disease 2019 (COVID-19). All infection experiments were performed under biosafety level 3 (BSL-3) containment conditions with enhanced respiratory personal protection equipment. The virus was propagated in HEK 293 cells at 90% confluency for 48 h. Cell supernatant was collected after infection and clarified by centrifugation at 200× *g* for 10 min. The supernatant was used as the virus stock, aliquoted, and stored at −80 °C. Virus stocks were quantified by titration.

### 2.3. Preparation of a Cell-Free Supernatant (CFS) from Lactobacillus sakei Probio65 (P65-CFS)

The CFS of *L. sakei* Probio65 (P65-CFS) was produced by culturing the bacteria in modified MRS broth at 37 °C for 24 h, which was then centrifuged at 3500 rpm for 15 min. The clear supernatant was collected, neutralized to pH 7.0, and filter-sterilized using a syringe filter (pore size 0.22 µm). Finally, the supernatant was concentrated by freeze-drying and reconstituted with sterile distilled water to 10% of its original volume. The extract was stored at −20 °C for subsequent analysis.

### 2.4. Cell-Viability Assay

The safety and cytotoxicity of P65-CFS to the human corneal epithelial (HCE), HEK 293, and Hela cell lines were tested using the MTT assay. In brief, each cell type was cultured in complete medium (Dulbecco’s Modified Eagle’s Medium (DMEM) supplemented with 10% fetal bovine serum (FBS) (HyClone Laboratories, Logan, UT, USA), penicillin (100 U/mL), and streptomycin (100 µg/mL), and incubated at 37 °C in a humidified atmosphere of 5% CO_2_. The cells were seeded in 96-well plates (4 × 10^4^ cells per well) and incubated for 24 h. Then, the medium was replaced with DMEM containing different concentrations of P65-CFS (50, 100, 150, 200, and 250 mg/mL) for 48 h at 37 °C and 5% CO_2_. Sterile PBS was used instead of P65-CFS in DMEM for the untreated control. At the end of the incubation period, the cells were washed in PBS, added to 50 μL of 5 mg/mL 3-(4,5-dimethylthiazol-2-yl)-2,5-diphenyl-2H-tetrazolium bromide (MTT) solution, and incubated for 45 min at 37 °C. All supernatant was aspirated; then, the purple formazan crystals were dissolved in isopropanol and the absorbance was measured at 570 nm. Percent viability was defined as the relative absorbance of P65-CFS-treated cells as compared to the control cells.

### 2.5. In Vitro Inhibition of SARS-CoV-2 Replication

Prior to infection, HEK 293 cells were seeded in 96-well plates at a density of 4 × 10^4^ cells per well, and incubated for 24 h. The attached cells were treated with 100 μL of the culture medium for virus infection (DMEM supplemented with penicillin (100 U/mL) and streptomycin (100 μg/mL) + P65-CFS (50, 100 and 150 mg/mL dissolved in DMSO to the final concentration of 0.1%) + SARS-CoV-2 virus (0.1 multiplicity of infection (MOI)). MOI was calculated based on the viral titer of SARS-CoV-2, which was quantified by RT-PCR as RNA copies/mL of the virus in respect to the number of cells seeded per well. For the positive control, the same amount of the virus was first heat-inactivated at 95 °C for 3 min prior to its addition to the cells. The virus was allowed to infect the cells for 2 h at 37 °C, and then the cells were rinsed three times with PBS to wash off any unabsorbed virus. Thereafter, the cells were continuously maintained in 100 μL of the same culture medium containing the P65-CFS in the absence of virus for 48 h. Positive and negative control samples were inoculated in the same manner, but the P65-CFS was replaced with 0.1% DMSO (vehicle) and phosphate-buffered saline (PBS), respectively [15]. The viral RNA was extracted from the SARS-CoV-2-infected HEK 293 cell supernatant using TRIzol reagent (Thermo Fisher, Carlsbad, CA, USA) according to the manufacturer’s instructions. The virus yield was measured using the quantitative real-time PCR method.

### 2.6. Measurement of Intracellular Reactive Oxygen Species (ROS)

Virus-induced changes in intracellular ROS levels were quantified using a DCFDA/H2DCFDA cellular oxidation kit, as previously described [16]. HEK 293 cells were infected with SARS-CoV-2 and treated with P65-CFS in the same manner as described in the viral infection scheme in this study. At the end of treatment, cells were washed twice with DMEM and incubated with 10 µM 2′-7′-Dichlorodihydrofluorescein diacetate (DCFH-DA, Sigma Aldrich, St. Louis, MO, USA) in DMEM for 30 min at 37 °C in the dark. The cell culture plates were photographed under a fluorescence microscope and the fluorescence intensity was quantified using Image J.

### 2.7. Phosphorylated Extracellular Signal-Regulated Kinases (p-ERK) Expression Using Immunocytochemistry

SARS-CoV-2-infected and P65-CFS-treated HEK293 cells were washed three times in PBS and fixed in 4% formaldehyde for 10 min. The cell membranes were permeabilized using 0.2% Triton X for 15 min, added to an image-iT FX signal enhancer, and then blocked with 5% goat serum diluted in 1X PBS. Cells were incubated at 4 °C with a primary antibody specific for p-ERK (1:100) for 12 h, after which the cells were incubated with secondary antibodies for another 1 h. Three PBS washes were performed between every step. The cell nuclei were counterstained with DAPI (Sigma Aldrich, St. Louis, MO, USA), washed with PBS, and finally photographed with a fluorescent microscope equipped with a digital camera (Nikon, Tokyo, Japan).

### 2.8. Post-Viral Infection Inflammatory Gene Expression by Quantitative Real-Time PCR

Total RNA was extracted from SARS-CoV-2-infected and P65-CFS-treated HEK293 cells using Trizol reagent (Invitrogen, Carlsbad, CA, USA). NanoDrop^TM^ (Thermo Fisher Scientific, Wilmington, DE, USA) was used to determine the RNA concentration and purity prior to the RT-PCR experiment. Briefly, 1 µg of RNA was added to a reaction mixture in a total volume of 25 µL containing random primers, and reverse-transcribed at 72 °C for 5 min and 37 °C for 60 min. The resulting cDNA was used in the real-time PCR experiment by adding 2 μL of the cDNA, 10 pmol of each gene-specific primer (primer sequences used were adapted from our previous study, as detailed by Rather et al. (2021)), and the Power SYBR^®^ Green PCR Master Mix (Applied Biosystems, Waltham, MA, USA) [12]. The expression levels were quantified using a 7500 real-time PCR system (Applied Biosystems, Waltham, MA, USA) according to the manufacturer’s recommendations; melting curve analysis was performed at the end of the cycling protocol. The GAPDH gene was used as an endogenous control, and the relative mRNA expression levels were determined using the 2^−ΔΔCT^ method.

## 3. Results

**Figure 1:**
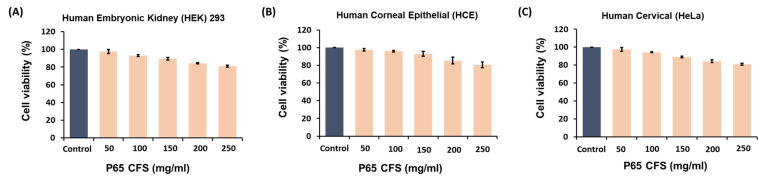
Cytotoxicity of the cell-free supernatant of *L. sakei* Probio65 (P65-CFS) for human cell lines, assessed by the MTT assay. (**A**) Human embryonic kidney (HEK) 293, (**B**) human corneal epithelial (HCE), and (**C**) human cervical (HeLa) cells were used to determine the toxicity of P65-CFS in a range of increasing concentrations. Cells were seeded in 96-well plates at a density of 4 × 10^4^ cells/well, then treated with P65-CFS for 24 h; the percentage of viability was determined in relation to an untreated control. Analysis of each sample was performed in triplicate, and data are represented as the means ± SD of three independent experiments.

We investigated the cytotoxic effects of P65-CFS on the human embryonic kidney (HEK) 293 cells, human corneal epithelial (HCE) cells, and human cervical (HeLa) cells at varying concentrations using the MTT assay (Figure 1). Increasing concentrations of P65-CFS reduced the viability of cells in all three cell lines; HCE cells were the most able to withstand these cytotoxic effects at 150 mg/mL, as their viability was only reduced by 7.0%. When treated at the highest concentration of 250 mg/mL of P65-CFS, HEK 293, HCE, and HeLa cells showed 81%, 80.67%, and 81% cell viability, respectively. The results were statistically insignificant for the untreated control cells, indicating that the P65-CFS did not display toxicity against human cells and the extract is safe.

**Figure 2:**
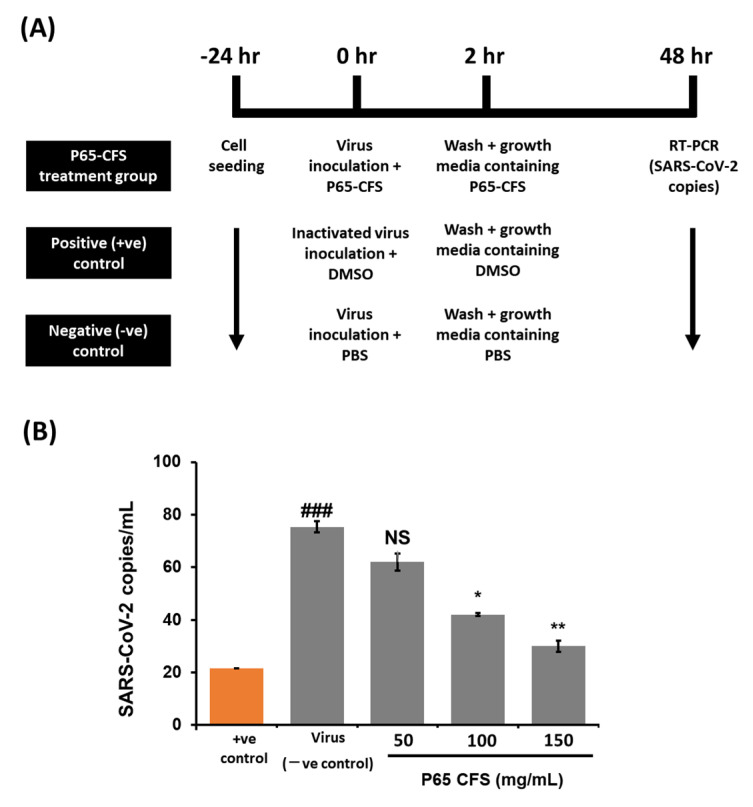
SARS-CoV-2 infection assay and the inhibitive effect of a cell-free supernatant of *L. sakei* Probio65 (P65-CFS) on viral replication. (**A**) Schematic of the SARS-CoV-2 infection assay and treatment groups. (**B**) Viral replication of SARS-CoV-2 in HEK293 quantified by RT-PCR. All wells were treated with an active virus, except for the positive controls, where the viruses were heat-inactivated at 95 °C for 15 min prior to their addition to cells. Cells were treated with P65-CFS (50–150 mg/mL) during the infection and after the infection; meanwhile, the positive control (+ve control) samples were treated in the same manner with the same volume of DMSO and the negative control (−ve control) samples were treated with PBS. Analysis of each sample were performed in triplicate, and data are represented as the means ± SD of three independent experiments. The results are presented as average values with the standard error of means (error bars). Independent *t*-tests were performed to compare the data with controls, where (###) represents *p* < 0.001, as compared to the positive control; (**) represents *p* < 0.01, and (*) represents *p* < 0.05, as compared to the virus (infected negative control).

Once it had been proven safe, the inhibitive effect of P65-CFS on SARS-CoV-2 replication was then evaluated by culturing the virus on HEK 293 cells at 0.1 MOI. According to the treatment scheme described in Figure 2A, the cells were incubated with the P65-CFS during a 2 h viral infection period, and the incubation continued for 48 h after the unabsorbed virus was washed off. The cell culture supernatants were collected at 48 h post-infection, and viral copies were determined using the RT-PCR method. Treatment with P65-CFS caused a notable dose-dependent inhibition of SAR-CoV-2 replication (Figure 2B). The lowest significantly effective concentration of P65-CFS that was used against SAR-CoV-2 replication was 100 mg/mL, where the viral copies were reduced by 44.24% as compared to the untreated infected control. At an increased concentration of 150 mg/mL, viral replication was reduced to 60.16%. As 150 mg/mL of P65-CFS did not exhibit a toxic effect on HEK 293 cells, given that they maintained a viability of 89.33% (Figure 1A) and showed the highest antiviral replication effect, the same concentration was evaluated in the following experiments.

**Figure 3:**
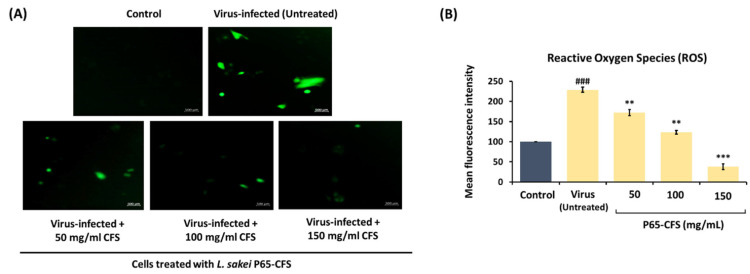
Accumulation of cellular reactive oxygen species (ROS) in HEK 293 cells upon infection with SARS-CoV-2. (**A**) Representative fluorescence images of ROS staining with the fluorogenic dye dichlorofluorescein (H2DC FDA), and (**B**) intensity quantitation by image J. At the end of the treatment, SARS-CoV-2-infected cells were stained with H2DCFDA for 30 min, washed with PBS, and imaged under a confocal microscope (scale is 500 µm). Analysis of each sample was performed in triplicate, and data are represented as the means ± SD of three independent experiments. Independent *T*-tests were performed to compare the data with the controls, where (###) represents *p* < 0.001 as compared to the control; (***) represents *p* < 0.001 and (**) represents *p* < 0.01 as compared to the virus (infected, untreated negative control).

Coronaviruses induce mitochondrial stress in the infected cells, which exacerbates the intracellular accumulation of reactive oxidizing species (ROS) and subsequently cell damage. In this study, when the HEK293 cells were infected with SARS-CoV-2, ROS were evidently accumulated in the intracellular compartment, as indicated by the green fluorogenic dye dichlorofluorescein (H2DCFDA) (Figure 3A). Using image J, the intensity of the fluorogenic dye, which correlates to the amount of ROS, was quantified (Figure 3B). Upon infection, the HEK293 cells developed a 129% increase in intracellular ROS, as compared to the naïve control. However, intracellular ROS were reduced when the infected cells were simultaneously treated with P65-CFS. The intracellular ROS were reduced by 24.76%, 46.16%, and 83.40% when the cells were treated with 50, 100, and 150 mg/mL P65-CFS, respectively (Figure 3B).

**Figure 4:**
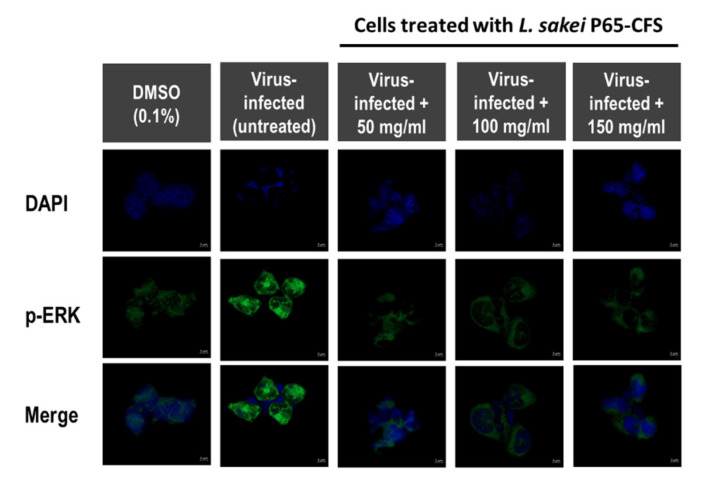
Expression of phosphorylated extracellular signal-regulated kinase (p-ERK) in HEK293 cells after infection with SARS-CoV-2 at MOI 0.1. During and after viral adsorption for 2 h, cells were treated with 50, 100, and 150 mg/mL of *L. sakei* Probio65 cell-free supernatant (P65-CFS). DMSO (0.1%) was used as positive control, while negative control cells were only treated with PBS. Cells were fixed 12 h after infection and double immunofluorescence staining was performed. The expression of p-ERK was visualized using confocal microscopy. Cell nuclei are stained blue, and p-ERK is stained green (scale bar = 5 μm).

Extracellular signal-regulated kinases (ERK) are one of the three main groups of mitogen-activated protein kinases (MAPKs), which regulate cell cycles, gene transcription, and post-transcriptional regulation. During coronavirus infection, they play an important role in apoptosis and cell survivability [17,18]. The MAPK/ERK pathway is the target of many viruses because it is one of the most important pathways in controlling cell growth. As shown in Figure 4, in virus-infected HEK 293 cells, p-ERK was highly expressed, as compared to the basal p-ERK levels (cells treated with 0.1% DMSO). It was observed that the addition of 50 mg/mL P65-CFS significantly weakened the intensity of p-ERK in SARS-CoV-2-infected cells. However, increasing the P65-CFS concentration to 100 and 150 mg/mL did not further reduce the p-ERK signal level in SARS-CoV-2-infected cells.

**Figure 5:**
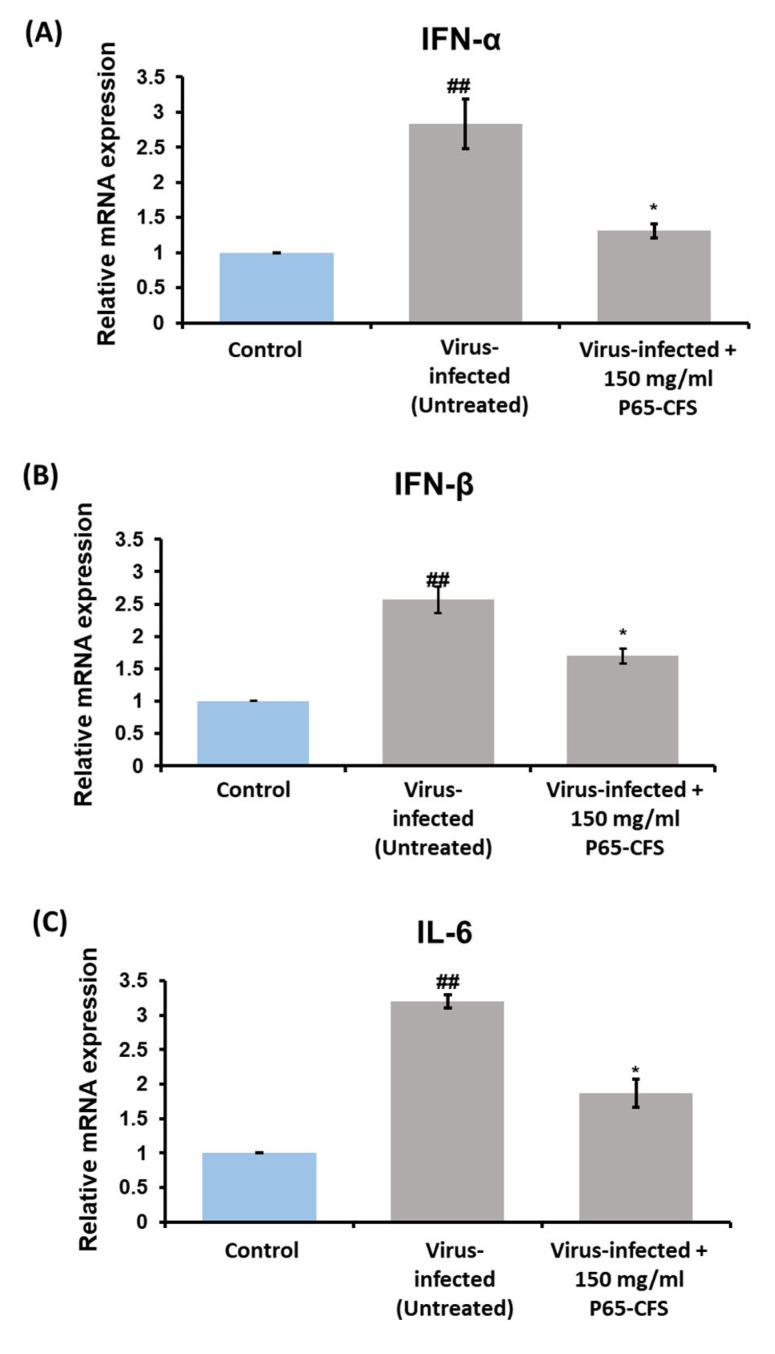
Modulation of the expression of inflammatory markers in HEK 293 cells after infection in the presence and absence of *L. sakei* Probio65 cell-free supernatant (P65-CFS). The expression of (**A**) IFN-α, (**B**) IFN-β, and (**C**) IL-6 were measured using quantitative PCR and normalized to GAPDH. All the experiments were conducted in triplicate and statistical analysis were performed using the *t*-test. Analysis of each sample was performed in triplicate, and data are represented as the means ± SD of three independent experiments. * *p* < 0.05 compared with virus-infected cells; ## *p* < 0.05 compared with the control.

Cytokine activation is one of the most prominent features of a cellular viral infection, as it is a means by which the cells stimulate downstream antiviral signaling pathways to counteract the virus [19]. In this study, the gene expression levels of cytokines IFN-α, IFN-ß, and IL-6 were determined after the cells were infected with SARS-CoV-2. The results showed that the cells expressed a 2.83-, 2.6-, and 3.2-fold increase in IFN-α, IFN-ß, and IL-6, respectively, as compared to the healthy cells (*p* < 0.05) (Figure 5). The treatment of P65-CFS at 150 mg/mL reduced the expression of IFN-α by 53.76%, IL-6 by 40.6%, and IFN-ß by 34.6%, as compared to the infected cells (*p* < 0.05) (Figure 5).

## 4. Discussion

While COVID-19 may now seem to have transitioned into a much more controllable phase, compared to the last three years, it is now a major global public health burden. This burden disproportionately affects the elderly population, who are at increased risk of severe disease, hospitalization, and death. We believe that there is still an urgent need for the development of new therapeutics that are safe, relatively cheap, and easily distributable to a wide range of populations. Our aim was to investigate the possibility of natural alternatives, such as probiotics, which have been known for decades to possess immunomodulating and antiviral properties. Probiotics have also been well reported as a therapeutic agent or adjuvant for vaccines in treating viral infections [20].

In this study, we observed that treatment using the extract of *L. sakei* Probio65, at a concentration that was not cytotoxic to the HEK 293 cells, significantly reduced SARS-CoV-2 replication by up to about 60%. Our results showed that this reduction in virus replication is accompanied by a concomitant reduction in ROS production. ROS play an important role in regulating the expression of some transcription factors and signal transduction molecules in living organisms. While they are important for cell cycle replication, ROS are considered damaging if they are produced in excessive amounts. During SARS-CoV-2 infection, viral proteins induce endoplasmic reticulum (ER) stress and mitochondrial dysfunction, which eventually triggers increased levels of ROS through inflammasomes [21,22].

As ROS continue to accumulate, the redox balance becomes disorganized, activating a series of immune responses. In particular, ROS have been reported to activate the STAT/IL-6 axis, which is responsible for regulating the release of cytokines to the site that is inflamed or has been exposed to the virus [22]. In most cases of COVID-19 infection, elderly individuals are more prone to develop a more severe response [23,24]. It has been proposed that this trend arises from the gradual decline in our ability to maintain redox homeostasis as we age, thereby increasing the risk of a cytokine storm and tissue injury [22]. Therefore, potential therapeutics aimed at inhibiting ROS-mediated cell damage are important if we are to prevent the worsening of the disease’s severity.

The increased levels of ROS during viral infection have also been reported to interfere with the ERK (extracellular signal-regulated kinase) pathway, which regulates vital cellular functions such as cell cycle progression, gene transcription, and post-transcriptional regulation [25]. The ERK pathway is also manipulated by extracellular ligands, such as protein components of viruses, and transmits cellular messages to the nucleus. Many viruses are known to induce cellular signaling via the ERK pathway, and the differential utilization of the ERK signaling pathway by different viruses shows the importance of this pathway in regulating a wide variety of cellular functions that ultimately influence viral infection [26]. Upon hijacking the host cells, viruses manipulate the ERK signaling pathway so that it will not be detected by the cell’s defense system, thus maintaining a proliferating state, and preventing the activation of cell apoptosis [27]. The SARS-CoV viral spike protein (S) was found to stimulate the expression of cyclooxygenase-2 (COX-2), which is involved in inflammation and is regulated by cytokines [28]. The S protein modulates precursors that activate the ERK pathway, which subsequently causes the expression of COX-2. This substantiates the claim that the activation of the ERK pathway is a pathogenesis feature of SARS-CoV-2 [28]. Our study proved that *L. sakei* Probio65 successfully lessened ERK phosphorylation, suggesting that the extract has the ability to affect the virus–cell signaling process during infection.

During a viral infection, our innate immunity plays an essential role as the first line of defense in sensing and activating antiviral mechanisms. As soon as an infection takes hold, the pattern-recognition receptors (PRR) detect the viral components and stimulate cytokine production as part of the innate immune response of the cells [29,30]. Among these cytokines, type-1 interferons, which include IFN-α, IFN-ß and IL-6, are promptly and transiently produced to contribute to the host’s antiviral defense; thus, the expression of these genes is also an indicator of the extent of the viral infection [29,31]. Upon the secretion and binding of these cytokines to their receptors, the Janus-activated kinases (JAK) are triggered, and eventually cause the cascading phosphorylation of signal transducers and activators of transcription (STATs), thereby facilitating the secretion of antiviral genes and other proinflammatory cytokines. Even though the activation of innate immunity and the secretion of proinflammatory cytokines are crucial for viral clearance, overactivation can lead to the excessive production of these cytokines, a condition known as a cytokine storm; this can result in multiple organ failure and death, as observed in many fatal COVID-19 cases [30]. In other words, inhibiting viral replication is crucial, but the regulation of the cascading innate immune response to prevent over-inflammation is equally important. Therefore, anti-cytokine therapy has become another key treatment strategy for patients with severe COVID-19. In the current study, treatment with the extract of *L. sakei* Probio65 led to the reduced expression of these cytokines in comparison to the SAR-CoV-2-infected cells, suggesting that the extract prohibited viral replication in the cells, and could also regulate the homeostasis of immune activation during viral infection.

The SARS-CoV-2 strain used in this study was not sequenced. However, the focus of this study was on the replicative effect of the virus on the host’s cells, and we believe the objective of the study was achieved without the strain being sequenced. However, at present, new variants are emerging due to mutations on the spike proteins, which are more closely related to the virus’s attachment to and entry into the host cells, and were not the focus of our study. Additionally, there are no significant differences between the results of this study and those of our previous study [12], except that, in our previous study, the antiviral effect was due to plantaricin E and F produced by *Lactobacillus plantarum* Probio-88. In the current study, meanwhile, the chemical composition of the extract from *L. sakei* Probio65 has not yet been elucidated. Nevertheless, *L. sakei* Probio65 is a safer and well-characterized probiotic strain.

## 5. Conclusions

In light of the continued prevalence of COVID-19 and the emergence of new variants, such as the XBB subvariant of Omicron detected in Saudi Arabia in October 2022, it is essential to develop potent antiviral compounds for use against SARS-CoV-2. This study highlights the therapeutic efficacy of *L. sakei* Probio65 for COVID-19 through its inhibition of viral multiplication and also of the subsequent pathogenicity. Our findings support its potential as a microbiome drug that might be used to prevent severe COVID-19 infection and to shorten the duration of infectiousness; it may therefore be useful as an adjuvant along with the currently available treatments.

## Data Availability

The data available has been presented in the paper.
